# Poor Health Related Quality of Life and Unhealthy Lifestyle Habits in Weight-Loss Treatment-Seeking Youth

**DOI:** 10.3390/ijerph18179355

**Published:** 2021-09-04

**Authors:** Enza Mozzillo, Eugenio Zito, Valeria Calcaterra, Nicola Corciulo, Mario Di Pietro, Anna Di Sessa, Roberto Franceschi, Maria Rosaria Licenziati, Giulio Maltoni, Giuseppe Morino, Barbara Predieri, Maria Elisabeth Street, Giuliana Trifirò, Francesca Gallè, Adriana Franzese, Giuliana Valerio

**Affiliations:** 1 Department of Translational Medical Science, Section of Pediatrics, Regional Center of Pediatric Diabetes, University of Naples Federico II, 80131 Naples, Italy; mozzilloenza@gmail.com (E.M.); franzese@unina.it (A.F.); 2 Department of Social Sciences, University of Naples Federico II, 80138 Naples, Italy; e.zito@unina.it; 3 Pediatrics and Adolescentology Unit, Department of Internal Medicine, University of Pavia, 27100 Pavia, Italy; valeria.calcaterra@unipv.it; 4 Department of Pediatrics, “Vittore Buzzi” Children’s Hospital, 20154 Milan, Italy; 5 Unit of Auxology and Pediatric Endocrinology, Sacred Heart of Jesus Hospital, Gallipoli, 73014 Lecce, Italy; n.corciulo57@gmail.com; 6 Pediatrics Unit, Hospital of Atri, 64032 Atri, Italy; mariodipietro16@gmail.com; 7 Department of Woman, Child, and General and Specialized Surgery, University of Campania “Luigi Vanvitelli”, 80138 Naples, Italy; anna.disessa@unicampania.it; 8 Pediatric Unit, S. Chiara Hospital, 38122 Trento, Italy; roberto.franceschi@apss.tn.it; 9 Obesity and Endocrine Disease Unit, Department of Neurosciences, Santobono-Pausilipon Children’s Hospital, 80129 Naples, Italy; mrlicenziati@gmail.com; 10 Pediatric Unit, IRCCS Azienda Ospedaliero-Universitaria di Bologna, 40138 Bologna, Italy; giulio.maltoni@aosp.bo.it; 11 Research Unit for Multifactorial Diseases, Bambino Gesù Children’s Hospital, 00165 Rome, Italy; giuseppe.morino@opbg.net; 12 Department of Medical and Surgical Sciences of the Mother, Children and Adults—Pediatric Unit University of Modena and Reggio Emilia, 41124 Modena, Italy; barbara.predieri@unimore.it; 13 Department of Mother and Child, Azienda USL-IRCCS di Reggio Emilia, 42123 Reggio Emilia, Italy; MariaElisabeth.Street@ausl.re.it; 14 Endocrinology and Metabolism Division, IRCCS Policlinico San Donato, 20097 Milan, Italy; gtrifi@libero.it; 15 Department of Movement Sciences and Wellbeing, Parthenope University of Naples, 80133 Naples, Italy; francesca.galle@uniparthenope.it

**Keywords:** adolescents, KIDMED score, mediterranean diet, obesity, overweight, physical activity, physical functioning, psychological functioning

## Abstract

Obesity is associated with unhealthy lifestyle behaviors and poor Health Related Quality of Life (HRQOL). The cumulative effect of lifestyle behaviors on HRQOL has been demonstrated in chronically ill adolescents, but not in adolescents with obesity. The present study aimed to assess the association between HRQOL and adherence to the Mediterranean Diet (MD) and/or low levels of physical activity (PA) in a large sample of outpatient adolescents with overweight or obesity seeking weight loss treatment. Four-hundred-twenty participants were enrolled from 10 Italian outpatient clinics. The demographics and anthropometric features, KIDMED scores, and exercise levels of the participants were collected, together with parental features. The HRQOL was assessed by the Pediatric Quality of Life Inventory (PedsQL™), Adolescents Version 4.0. PedsQL total score and functioning subscales were lower in adolescents who reported one or two unhealthy habits. Compared with the high/intermediate groups, the risk of low HRQOL was twice as high for each unit increase in BMI SDS, while the percentage was reduced by 12.2% for every unit increase in the KIDMED score and by 32.3% for each hour increase of exercise. The clustering of these two unhealthy behaviors conferred a 120% higher risk of low HRQOL. Similarly, adolescents displaying better diet quality and/or a physically more active lifestyle have better physical and psychological functioning. Further studies are needed to disclose whether these characteristics may be predictive of better adherence to weight loss treatment.

## 1. Introduction

Pediatric obesity, a multifactorial condition resulting from a complex interaction between individual, social, and environmental factors, represents a global public health problem [1]. Energy balance related behaviors leading to increased weight gain, such as inactivity and unbalanced diet, typically initiate in childhood, are reinforced during adolescence, a period of substantial physical, social and psychological changes, and are maintained throughout adulthood [2]. The literature has shown that these unhealthy behaviors are likely to cluster in the same individual [3] and exert their additive effect on the risk of pediatric obesity [4]. Parental characteristics (i.e., weight status, education, and socioeconomic level) are also important risk factors for children’s overweight and obesity [5,6,7].

The prevalence of severe obesity is increased in pediatric age [8,9,10] and it is associated with various complications, which undermine not only physical health but also psychological and social wellbeing [11]. Specifically, youth who are obese may experience peer victimization/bullying, poor self-esteem, dissatisfaction with body image, difficulties in interpersonal relationships or social isolation, behavioral problems, depression or anxiety [12]. These psychosocial consequences may significantly impact school achievement, attendance, behavior in school, and limit sports participation [13,14].

According to the WHO definition, health is not simply the absence of disease but rather a condition of complete bio-psycho-social wellbeing [15]. The Health Related Quality of Life (HRQOL) [16] is a global measure of perceived health and burden of a disease. The HRQOL, a multidimensional and subjective measure of an individual’s health, encompasses physical, emotional, and social wellbeing and is used to assess the individual’s perceptions of the impact of their disease [17].

A significant and negative impact of obesity on HRQOL has been demonstrated in adults [18] as well as in children [19]. Indeed, systematic reviews and meta-analyses have shown that children and adolescents with obesity have lower HRQOL scores compared to youths with healthy weight [19]. This is particularly evident in the industrial societies [19,20,21,22,23,24,25].

Few studies have focused on the link between HRQOL in adolescents and health related behaviors, such as an adherence to the Mediterranean diet (MD) or the levels of physical activity (PA) [26,27]. A positive relationship between healthy diet or high PA levels and physical health or psychosocial wellbeing has been reported in adolescents [28,29]. Adolescents spending more time in PA and less time in screen viewing showed higher HRQOL scores, particularly in physical and social domains [30].

However, few studies have analyzed the influence of the cumulative effect of obesogenic lifestyle behaviors in adolescents with a chronic disease [31]. In particular, the association between individual or clustered unhealthy lifestyle habits and HRQOL has been little investigated in adolescents with obesity. Therefore, our aim was to evaluate the variables associated with the self-reported and parent-proxy reported HRQOL, such as gender, body mass index (BMI), dietary behaviors, PA levels, and parental BMI and education level in a large sample of Italian adolescents seeking weight loss treatment in outpatient clinics of the Italian Society of Pediatric Endocrinology and Diabetology (ISPED). The combined effect of unhealthy behaviors on the HRQOL was also assessed.

## 2. Materials and Methods

This cross-sectional multicenter study was carried out by the Childhood Obesity study Group of the Italian Society for Pediatric Endocrinology and Diabetology (ISPED). Participants were represented by youths seeking for weight loss treatment; they were recruited in 10 outpatient clinics for the care of Pediatric Obesity at community or university hospitals throughout the Italian country. The inclusion criteria were age 13.0–17.0 years; Caucasian ethnicity; overweight or obesity; first visit at the outpatient clinic. The exclusion criteria were secondary causes of obesity (genetic, endocrine or iatrogenic forms); presence of other chronic diseases or mental illness. Four-hundred-twenty adolescents (187 boys, 233 girls; mean age 14.2 ± 1.1 years; range 13–17) were consecutively enrolled over a 12-month period (January–December 2017). Demographic data (age and gender) were collected from medical records. The height and weight of the adolescents were measured by the same investigator in each center. Height was measured to the nearest 0.1 cm with a wall-mounted stadiometer, while weight was determined to the nearest 0.1 kg on a medical scale. The BMI was calculated (weight/height^2^) for both parents and adolescents. Furthermore, the BMI standard deviation score (BMI-SDS) was computed in adolescents as measure of relative BMI adjusted for age and sex. Adolescents were asked to answer on their lifestyle habits (adherence to the MD, PA levels). Parents were asked to report their own weight and height and the highest grade and year of education attained according to the Italian education system [elementary (five years), middle school (three years), high school (five years), degree (over three years)]. Adolescents’ self-reported and parent-proxy reported data about HRQOL were collected by an ad hoc questionnaire.

The research protocol was approved by the Ethics Committee of the University of Naples Federico II, coordinating center of the study (protocol number 88/16), and subsequently by the Ethics Committee of the other participating centers. Written informed consent was obtained from both the adolescents and their parents in accordance with the new version of the Helsinki Declaration. The data of participants and their parents were anonymously registered in a database using an alphanumeric and progressive identification code.

### 2.1. Dietary Behaviors

Dietary behaviors were assessed through the KIDMED questionnaire, which allows assessing a variety, or combination of different foods and beverage and the frequency with which they are habitually consumed, providing an estimate of an individual’s adherence to the MD pattern [32]. The KIDMED questionnaire is composed by the following 16 questions: (1) Takes a fruit or a fruit juice every day; (2) Has a second fruit every day; (3) Has fresh or cooked vegetables regularly once a day; (4) Has fresh or cooked vegetables more than once a day; (5) Consumes fish regularly (at least 2–3 times per week); (6) Goes more than once a week to a fast-food (hamburger) restaurant; (7) Likes pulses and eats them more than once a week; (8) Consumes pasta or rice almost every day (5 or more times per week); (9) Has cereals or grains (bread, crackers, etc.) for breakfast; (10) Consumes nuts regularly (at least 2–3 times per week); (11) Uses olive oil at home; (12) Skips breakfast; (13) Has a dairy product for breakfast (yoghurt, milk, etc.); (14) Has commercially baked goods or pastries for breakfast; (15) Takes two yoghurts and/or some cheese (40 g) daily; (16) Takes sweets and candy several times every day. These questions were posed by the same pediatrician to each participant through an interview. For each “yes” response, one point was given to answers representing positive food habits (items 1–5, 7–11, 13, 15), and one point was subtracted for those representing negative food habits (items 6, 12, 14, 16). Two categories of adherence were defined according to a score ≤3 (poor adherence) and ≥4 (average/good adherence) [32].

### 2.2. Physical Activity

PA habits were examined by asking the weekly average amount of hours spent for sports (except for physical education at school) or other structured types of PA (dancing, formal exercise programs or use of fitness centers). Adolescents who exercised <3 h/week were classified as less active, while adolescents engaged in ≥3 h of exercise per week were classified as “active” [33].

### 2.3. Clustering of Unhealthy Lifestyle Habits

The clustering of unhealthy lifestyle habits was calculated by adding for each participant the number of unhealthy behaviors adopted, namely low MD adherence indicated by a KIDMED score ≤3 [32] and low PA expressed by less than 3 h of regular exercise per week [33].

### 2.4. Health Related Quality of Life

HRQOL was measured by using the Pediatric Quality of Life Inventory (PedsQL™), Italian Version 4.0 for adolescents (13–18 years old) [16,34]. This is a generic tool and allows a parallel adolescent self-report and a parent proxy-report. The multidimensional instrument PedsQL is a validated 23-item scale comprising four subscales—Physical Functioning (8 items) (e.g., problems with running, lifting something heavy), Emotional Functioning (5 items) (e.g., feeling afraid, trouble sleeping), Social Functioning (5 items) (e.g., getting along with other children), and School Functioning (5 items) (e.g., paying attention in class). Answers were scored along a 5-point Likert-type scale: never (0), almost never (1), sometimes (2), often (3), or almost always (4). Items were reversely scored and transformed to a 0–100 (0 = 100, 1 = 75, 2 = 50, 3 = 25, 4 = 0) scale so that higher scores reflected better HRQOL, as per scoring guidelines. Subscale scores were computed from sum and average of the total items within each subscale. The Total Functioning Score is the sum and average of all items across the four subscales. We created three levels of total functioning score based on tertiles of the total score obtained in our population of adolescents: high functioning ≥73.3, intermediate functioning between 73.2 and 68.4 and low functioning ≤68.5.

### 2.5. Statistical Analyses

Statistical analyses were performed using SPSS for Windows, version 25.0 (SPSS Inc., Chicago, IL, USA). A two-sided *p* value of ≤0.05 was considered statistically significant in all analyses. All the variables were not normally distributed and were expressed as medians (25th–75th percentile); categorical data were presented as absolute frequencies and percent values.

The Mann–Whitney U test was used to compare variables between two groups (i.e., males vs. females), while the Wilcoxon Rank-Sum Test was used to compare children and parent scores on the PedsQL 4.0 Generic Core Scales. The Kruskal–Wallis H test was used to compare groups stratified according to tertiles of HRQOL scores or the clustering of unhealthy lifestyle habits; a Mann–Whitney U test with a Bonferroni correction was performed to ascertain which pairs of groups differed significantly from one another. Chi-squared test was used to compare proportions. Intra-class correlation coefficients (ICCs) were utilized to evaluate agreement between patient self-report and parent proxy-report on the PedsQL scales (ICCs ≤ 0.40 poor to fair agreement, 0.41–0.60 moderate agreement, 0.61–0.80 good agreement, and 0.81–1.00 excellent agreement). The Eta squared (small effect 0.01, medium 0.06, large 0.14) or the Cramer’s V (no or little effect <0.1, low 0.1–0.3, moderate 0.3–0.5, high >0.5) were calculated as measures of effect size for these comparisons [35].

Two models of multiple logistic regression analysis were applied to calculate the Odds Ratios (ORs) and 95% Confidence Intervals (CIs). In both models the low functioning total score of PedsQL was the dependent variable and the intermediate/high functioning was the reference category. In the first model the independent variables were represented by BMI-SDS, parents’ education level, KIDMED score and PA levels; in the second model variables related with diet and PA were replaced with clustering of unhealthy lifestyle habits in order to assess their possible cumulative role in determining HRQOL.

## 3. Results

The demographic and anthropometric features, lifestyle habits, parental characteristics, and PedsQL scores of the total sample and stratified by gender are presented in Table 1.

Self-report forms of the PedsQL were completed by 420 adolescents and proxy-reports forms were completed by 413 parents. Girls were slightly older than boys. No differences were found between genders in the total PedsQL score and subscales, except for emotional functioning, which was lower in girls. Similar data were registered for the emotional functioning reported by parents (Table 1). However, the effect size of these differences was small. Across all PedsQL scales, adolescents self-reported significantly higher scores than their parents, with the most significant differences (*p* < 0.001) related with total functioning, physical functioning and social functioning both in the whole sample and by gender (Table 1).

ICCs between adolescent self-report and parent proxy report across the PedsQL 4.0 Generic Core Scales are presented in Table 2. The ICCs were in the good agreement range; the greatest agreement was found for the total score, whereas the lowest on emotional functioning.

Adolescents within the low total functioning HRQOL showed higher BMI-SDS, and lower KIDMED score, weekly hours of exercise, father’s and mother’s education level compared with youths with intermediate and/or high functioning HRQOL (Table 3). The effect size was small for all these differences but for weekly hours of exercise.

Considering the cluster of unhealthy lifestyle habits, 79 adolescents (18.8%) reported no unhealthy lifestyle component, 236 (56.2%) one unhealthy habit and 105 (25.0%) two unhealthy habits. No differences were found regarding gender distribution, age, BMI, and BMI-SDS among these groups, while adolescents reporting two unhealthy lifestyle habits presented significantly lower father’s and mothers’ education level than those with no unhealthy component (Table 4). All the PedsQL scores (total and subscales) significantly decreased with the increase of one or two unhealthy habits. The effect size was medium for total scores and physical functioning in both adolescents and parents, medium for social functioning in the youths and small for the other domains.

Multiple logistic regression analyses showed that, when the unhealthy behaviors were considered separately, low total functioning was positively associated with BMI-SDS and negatively associated with KIDMED score and weekly hours of exercise, while the association with parents’ education level was not confirmed. When the clustering of unhealthy habits was included in the model in place of the single components, adolescents within the low functioning scores showed a twofold risk of having low HRQOL total score compared with their respective counterpart with high/intermediate functioning (Table 5).

## 4. Discussion

The present study demonstrated that severity of obesity, adherence to the MD, and levels of exercise were independently associated with worse HRQOL in a population of outpatient adolescents seeking weight loss. HRQOL total score decreased as the number of unhealthy components increased. Clustering of unhealthy habits conferred a twofold risk of low functioning compared with adolescents with high/intermediate functioning.

The adverse consequences of pediatric obesity on physical and psychosocial health and their effects on the HRQOL are widely acknowledged [19,36,37]. Most dimensions of HRQOL are consistently affected in overweight and obese children and adolescents compared to normal-weight youths [19]. Among the different dimensions of the PedsQL, the emotional functioning appeared to be more affected in obese youths recruited from clinical setting than from general population [19] both in the self-reported and the parent-proxy reported studies. Previous studies [19] showed gender-related differences in HRQOL for social functioning, physical appearance, self-esteem, and total scores, with girls being generally more affected than boys [26]. In our sample of youths with obesity we found no gender-related differences in the HRQOL total score and in the other sub-domains, with the exception of emotional functioning that scored worse in girls than boys. Numerous studies have demonstrated that weight stigma is highly prevalent in individuals with obesity [38]. Adolescents with obesity experience higher rates of weight teasing or bullying [39] and may suffer of several psychosocial complaints [12]. Although adolescents of both genders are exposed to pressures to conform to an ideal body image, the lower score found in the emotional functioning in girls may reflect the fact that perception of body weight may be generally more strongly associated with low life satisfaction among girls compared to boys [40]. A relationship between body dissatisfaction and its effects on HRQOL may start very early in life, since it has been reported that girls as early as 6 years are already exposed to peers and media influence on body image and dieting awareness [41,42].

Despite the restricted range of BMI status (BMI-SDS between 1.9–2.7) in our sample, adolescents with lower functioning of the total HRQOL score showed a more severe degree of obesity compared with youths with intermediate and/or high functioning. This finding is in agreement with other studies performed in clinical samples [43,44], suggesting that the weight related differences, usually reported in the general population, may also occur among the very severely youths with obesity.

Combinations of unhealthy lifestyle behaviors are key elements for unfavorable weight gain in children and adolescents [45,46,47,48]. Several cross-sectional and longitudinal studies have reported a positive association between individual lifestyle behaviors and physical or mental health in adolescents [26,49,50]. We found that adolescents within the lower total functioning of HRQOL showed lower KIDMED scores and more time spent in weekly exercise compared with youths with intermediate and/or high functioning. MD is one of the healthiest dietary models that shows benefits regarding life expectancy and cardiovascular diseases that are frequently related to obesity [51]. A great consumption of fruits and vegetables has been shown to have a beneficial impact on physical health through several pathways associated to numerous biologically active components [52]. Moreover, diet quality has a beneficial effect on self-perception and is associated with fewer externalizing problems that are usually linked to negative behaviors as being bullied [53,54]. Regarding lifestyle, the association between PA, sedentary behavior, and HRQOL in the general healthy population of children and adolescents has been systematically reviewed by Wu et al. [29]. Higher levels of PA and less sedentary behavior were associated with better HRQOL with a dose-response relationship. Of note, Gopinath et al. demonstrated that regular PA was prospectively associated with a higher perceived HRQOL in a cohort of Australian adolescents [30]. Unhealthy behaviors tend to be associated each other and may significantly predict global health. MD adherence and PA engagement were associated with better perception of physical and psychological wellbeing, and improved family relationships, autonomy support and perceptions of the school environment in Spanish adolescents [55]. A combination of unhealthy lifestyle behaviors and poor HRQOL was also reported by Gopinath et al. in Australian school children [56]. The combination of high levels of physical fitness and optimal adherence to Mediterranean diet was positively associated with better HRQOL scores in Portuguese adolescents [57].

As far as we are aware, no study has previously analyzed the association of unhealthy behaviors in adolescents with obesity and self-reported HRQOL. As it was reported in the general population, lower functioning of the total HRQOL score was independently associated with lower KIDMED score or weekly hours of exercise in our sample of obese adolescents. The risk of low HRQOL was twice as high for each unit increase in BMI SDS while the percentage was reduced by 12.2% for every unit increase in the KIDMED score and by 32.3% for each hour increase of exercise. The clustering of these two unhealthy behaviors conferred a 120% higher risk of low HRQOL.

Treating obese adolescents implies good adherence to lifestyle changes. Of note, among the determinants of weight loss, the participation in exercise groups before the beginning of weight loss treatment was a strong predictor of success [58,59]. Furthermore, several studies reported the beneficial effect of weight loss on the improved HRQOL [60,61], but the quality of life at baseline may also impact adherence and treatment outcomes in obese adolescents [62]. Therefore, a virtuous circle between healthy lifestyle and better HRQOL may have an impact on success of obesity treatment.

The median BMI of both parents in our study was in the overweight range. The strong association between parental weight status and childhood obesity [63] may imply that parents have a role in the development of child overweight and obesity through both genetics and shared environmental factors. At the same time, parents are also significant agents for change in the treatment of adolescent obesity [64,65] and their point of view on the physical and psychosocial implications related to obesity may provide complementary information to their child’s report. Therefore, we decided to assess both adolescent self-reports and a parent proxy-report of PedsQL. Adolescent-parent concordance was quite good in all the domains (ICC = 0.654–0.737), with the highest agreement on the total score and the lowest on the emotional functioning. Across all PedsQL scales, parents reported significantly lower scores than their children, predominantly on the total score and social health. Our findings are consistent with previous studies that showed that HRQOL scores were significantly lower on the parent reports than on the self-reports of obese youths for some or all of the dimensions studied [19]. Levels of agreement between adolescent self-report and parent proxy-reports on the PedsQLs can be affected by parents’ education levels or their own quality of life [66,67]. While we did not find any association between low total functioning and parents’ education level, we cannot exclude that parents with obesity themselves experienced negative influences on psychological aspects, such as self-esteem, body image, and emotional state, and projected these bad experiences onto their child’s experiences. Our findings suggest that parents’ distress and worry over the physical and psychosocial implications of overweight may account for overestimation of the effects of obesity on child’s physical functioning and other domains as the social and psychosocial ones [68,69,70]. Over the past 10 years, public health organizations from several European countries have been involved in policies to contrast obesity [71]. Thus, awareness about the causes and consequences of obesity in children is increased not only among health professionals, but also in the general public, including families and teachers. Discrepancies in the perceived HRQOL between parents of children have been also reported in several chronic diseases, such as type 1 diabetes or cystic fibrosis [31,72,73].

This study presents some limitations, such as the cross-sectional design and the lack of normal-weight control group, therefore the analyses of the total PedsQL score were done using tertiles calculated on our own population. Only participation to structured PA was considered, while other forms of incidental PA, such as daily activities at school, at home, or during transport were not assessed. This decision was driven by the evidence that estimate of PA levels is challenging in youths, specifically in those affected by obesity, who tend to overestimate PA [74]. We supposed that focusing the item only on programmed and regular physical activities could have reduced the bias. Lastly, no information was available on the quality of life of parents, which could have allowed to assess its possible modulation effect on the quality of life of their children. Instead, the strength of our study is the multi-centric design and the use of validated questionnaires to analyze diet quality and HRQOL. Moreover, the interpretation of the different levels of agreement between self-reported and parent proxy-reported HRQOL was based on a very high percentage of parents who participated (more than 98%).

## 5. Conclusions

The main objective of the treatment of pediatric obesity is a permanent change in the child’s eating habits and lifestyle, leading to a negative caloric balance and a progressive weight loss. Another important goal is improving mental health and HRQOL [2].

Our findings highlight for the first time the influence of the cumulative effect of two unhealthy behaviors, such as unhealthy diet pattern and low PA, on lower general health and physical functioning domains in adolescents with obesity.

The assessment of the HRQOL is crucial in the care of youth seeking weight-loss treatment. Promoting healthy eating behaviors and an active lifestyle in obese adolescents may positively impact their quality of life and establish a virtuous circle of successful treatment.

Further studies are needed to disclose whether the baseline lifestyle characteristics and the HRQOL score may be predictive of better adherence to weight loss treatment in adolescents. These data will be helpful to optimize the management and to apply personalized medicine paths in obesity treatment.

## Figures and Tables

**Table 1:** Participants’ demographic and anthropometric features, lifestyle habits, parental characteristics, and PedsQL scores of adolescents and parents of the total sample and stratified by gender.

	Total Group (*n* = 420)	Boys (*n* = 187)	Girls (*n* = 233)	*p*	Effect Size
Adolescents’ Features					
Age (year)	14.0 (13.2–15.0)	14.0 (13.0–14.8)	14.0 (13.2–15.3)	0.020	0.013 *^η2^*
Height (cm)	162 (157–168)	165 (159–171)	160 (155–165)	<0.001	0.076 *^η2^*
Weight (kg)	82.3 (73.6–93.2)	85.6 (74.2–100.7)	80.0 (73.3–90.0)	0.001	0.39 *^d^*
BMI (kg/m^2^)	31.2 (28.7–34.2)	31.9 (28.6–34.8)	31.0 (28.7–33.7)	0.604	0.001 *^η2^*
BMI-SDS	2.3 (1.9–2.7)	2.4 (1.9–2.8)	2.3 (1.9–2.7)	0.748	0 *^η2^*
KIDMED score	5 (3–7)	5 (3–6)	5 (3–7)	0.423	0.002 *^η2^*
Exercise (hours/week)	0 (0–3)	0 (0–3)	0 (0–2)	0.471	0.001 *^η2^*
Kidmed score ≤3 (*n*%)	135 (32.1)	62 (33.2)	73 (31.3)	0.691	0.019 ^V^
Exercise ≤3 h/week (*n*%)	311 (74.1)	135 (72.2)	176 (75.5)	0.437	0.038 ^V^
Parents’ Features					
Father’s BMI (kg/m^2^)	28.0 (25.7–31.8)	27.8 (25.7–32.3)	28.3 (25.6–31.8)	0.607	0.011 *^η2^*
Mother’s BMI (kg/m^2^)	28.1 (24.2–32.3)	27.9 (24.1–32.4)	28.2 (24.6–32.2)	0.469	0.006 *^η2^*
Father’s education level (year)	13 (8–13)	13 (8–13)	13 (8–13)	0.784	0.01 *^η2^*
Mother’s education level (year)	13 (8–13)	13 (8–13)	13 (8–13)	0.205	0.011 *^η2^*
Adolescents’ PedsQL					
Total functioning	75 (65–83) ***	76 (66–83) ***	74 (63–81) ***	0.183	0.004 *^η2^*
Physical functioning	75 (66–84) ***	78 (66–84) ***	75 (66–84) ***	0.152	0.005 *^η2^*
Emotional functioning	70 (55–80) **	75 (60–85) **	65 (50–80)	0.004	0.02 *^η2^*
Social functioning	85 (70–95) ***	85 (70–90) ***	85 (67–95) ***	0.455	0.001 *^η2^*
School functioning	70 (55–85) *	75 (50–85) *	70 (57–85) **	0.925	0 *^η2^*
Parent proxy PedsQL					
Total functioning	67 (54–78)	67 (57–79)	67 (52–75)	0.101	0.011 *^η2^*
Physical functioning	66 (53–81)	66 (53–84)	66 (50–81)	0.057	0.014 *^η2^*
Emotional functioning	65 (50–80)	70 (55–80)	60 (50–79)	0.019	0.019 *^η2^*
Social functioning	75 (55–90)	75 (55–90)	70 (50–90)	0.468	0.004 *^η2^*
School functioning	65 (50–80)	65 (50–80)	70 (50–80)	0.761	0.002 *^η2^*

Parent-proxy PedsQl was available in 413 parents. *^η2^* Eta squared; *^d^* Cohen’s *d*; ^V^ Cramer’s V. Mann–Whitney U test was used to compare gender groups; Chi-squared test was used to compare proportions between genders. Significant differences between genders are shown in the column. Wilcoxon Rank-Sum Test was used to compare adolescents’ self-reported scores and parent-proxy reported scores in the whole group and by gender: asterisks denote significant differences between adolescents’ and parents’ scores. *** *p* <0.001; ** *p* < 0.01; * *p* < 0.05.

**Table 2:** Intra-class correlation coefficients (ICC) between adolescent self-report and parent proxy-report.

	Intra-Class Correlation	Lower 95% CI	Higher 95% CI	*p*
Total functioning	0.737	0.681	0.783	<0.001
Physical functioning	0.685	0.617	0.740	<0.001
Emotional functioning	0.640	0.563	0.703	<0.001
Social functioning	0.654	0.580	0.714	<0.001
School functioning	0.666	0.595	0.725	<0.001

CI: Confidence intervals; ICCs are designated as 0.40 poor to fair agreement, 0.41–0.60 moderate agreement, 0.61–0.80 good agreement, and 0.81–1.00 excellent agreement.

**Table 3:** Demographic, anthropometric features and lifestyle habits, and parental characteristics among adolescents stratified by total HRQOL functioning.

	Total HRQOL Functioning				
	Low	Intermediate	High	*p*	Effect Size
N	136	134	150		
Sex (M/F)	57/79	55/79	75/75	0.241	0.082 ^V^
Age (yr)	13.2 (14.0–15.0)	14.0 (13.2–15.2)	14.0 (13.0–15.0)	0.522	0.003 *^η2^*
Height (cm)	1.62 (1.58–1.68)	1.62 (1.58–1.67)	1.61 (1.55–1.68)	0.588	0.002 *^η2^*
Weight (kg)	86.0 (76.6–95.9)	82.5 (73.6–92.1) ^a^	79.4 (70.6–90.0) ^b^	0.003	0.029 *^η2^*
BMI (kg/m^2^)	32.5 (29.5–36.4)	30.7 (28.2–33.8) ^a^	30.4 (28.5–33.3) ^b^	<0.001	0.048 *^η2^*
BMI-SDS	2.5 (2.1–2.9)	2.3 (1.8–2.6) ^a^	2.2 (1.8–2.6) ^b^	<0.001	0.042 *^η2^*
KIDMED score	4 (2–6)	5 (3–7) ^a^	5 (4–7) ^b^	0.001	0.035 *^η2^*
Exercise (hours/week)	0 (0–0.75)	0 (0–3) ^a^	2 (0–3) ^b^	<0.001	0.075 *^η2^*
Father’s BMI	28.7 (26.3–32.5)	27.9 (26.0–31.8)	27.7 (25.3–30.9)	0.233	0.008 *^η2^*
Mother’s BMI	29.3 (24.9–33.0)	27.8 (23.9–31.8)	27.1 (23.9–31.8)	0.102	0.007 *^η2^*
Father’s education level (year)	8 (8–13)	13 (8–13)	13 (8–13) ^b^	0.028	0.015 *^η2^*
Mother’s education level (year)	8 (8–13)	13 (8–13) ^a^	13 (8–13) ^b^	0.019	0.020 *^η2^*

^V^ Cramer’s V; *^η2^* Eta squared. Chi-squared test was used to compare proportions. Kruskal–Wallis *H* test was used to compare the overall *p* among the three groups of total HRQOL functioning. Overall significant differences among groups are shown in the *p* column. Mann-Whitney test with a Bonferroni correction was performed to ascertain the difference between each pair of groups of low, intermediate and high HRQOL: ^a^ significant differences between intermediate and low, ^b^ significant differences between high and low (*p* < 0.05).

**Table 4:** Demographic and anthropometric features of participants and adolescents’ self-reported and parent-proxy reported PedsQL scores by clusters of unhealthy lifestyle habits.

	Components of Unhealthy Lifestyle			*p*	Effect Size
	0	1	2		
Adolescents’ features					
N	79	236	105		
Sex (M/F)	41/38	95/141	51/54	0.124	0.1 ^v^
Age (year)	14 (13.2–15)	14 (13.1–15)	14 (13.2–14.8)	0.888	0.001 *^η2^*
Height (cm)	1.65 (1.59–1.70)	1.61 (1.55–1.67) ^a^	1.63 (1.58–1.68) ^b^	0.001	0.037 *^η2^*
Weight (kg)	85.4 (73.5–100.0)	81 (73.3–91)	85.5 (74.3–97.0)	0.165	0.012 *^η2^*
BMI (kg/m^2^)	31.0 (28.7–33.4)	31.4 (28.6–34.4)	31.4 (29.1–34.9)	0.430	0.004 *^η2^*
BMI-SDS	2.3 (1.9–2.6)	2.4 (1.9–2.7)	2.3 (1.9–2.8)	0.428	0.005 *^η2^*
Parents’ features					
Father’s BMI (kg/m^2^)	27.5 (25.0–31.6)	28.4 (25.5–32.4)	28.2 (26.4–31.6)	0.164	0.009 *^η2^*
Mother’s BMI (kg/m^2^)	28.1 (23.8–32.2)	28.0 (24.2–32.4)	28.3 (24.8–32.2)	0.921	0.001 *^η2^*
Father’s education level (year)	13 (8–13)	13 (8–13) ^a^	8 (8–13) ^c^	0.005	0.026 *^η2^*
Mother’s education level (year)	13 (9.2–13)	13 (8–13) ^a^	13 (8–13) ^c^	0.002	0.031 *^η2^*
Adolescents PedsQL					
Total functioning	80 (75–85)	74 (64–83) ^a^	70 (56–77)^bc^	<0.001	0.086 *^η2^*
Physical functioning	84 (78–91)	75 (66–84)^a^	72 (56–78) ^bc^	<0.001	0.086 *^η2^*
Emotional functioning	75 (60–85)	70 (55–80) ^a^	65 (50–80) ^c^	0.003	0.035 *^η2^*
Social functioning	90 (85–100)	85 (70–95) ^a^	75 (60–90) ^bc^	<0.001	0.063 *^η2^*
School functioning	75 (65–85)	70 (55–85)	65 (47–85) ^c^	0.013	0.026 *^η2^*
Parent-proxy PedsQL					
Total functioning	75 (62–84)	67 (54–77) ^a^	62 (48–74) ^bc^	<0.001	0.069 *^η2^*
Physical functioning	75 (63–88)	66 (50–81) ^a^	62 (44–72) ^bc^	<0.001	0.066 *^η2^*
Emotional functioning	70 (55–85)	65 (50–80)	60 (45–75) ^c^	0.016	0.022 *^η2^*
Social functioning	80 (65–95)	75 (55–90) ^a^	65 (45–84) ^bc^	<0.001	0.039 *^η2^*
School functioning	70 (6–90)	67 (50–80) ^a^	60 (41–75) ^bc^	0.003	0.032 *^η2^*

^V^ Cramer’s V; *^η2^* Eta squared. Chi-squared test was used to compare proportions. Kruskal–Wallis *H* test was used to compare the overall *p* among the three groups with and without unhealthy habits. Overall significant differences among groups are shown in the *p* column. Mann-Whitney test with a Bonferroni correction was performed to ascertain the difference between each pair of groups with none, one or two unhealthy habits: ^a^ significant differences between intermediate and low, ^b^ significant differences between high and intermediate, ^c^ significant differences between high and low (*p* < 0.05).

**Table 5:** Multiple logistic regression analyses of the associations between low total functioning and parents’ or adolescents’ characteristics (high/intermediate functioning was used as reference category).

	Dependent Variable Low Total Functioning (High/Intermediate Functioning as Reference Category)	
Independent Variables	Model 1	Model 2
BMI-SDS	1.907 (1.298–2.804) **	2.004 (1.372–2.927) **
Father’s education level (year)	1.000 (0.929–1.077)	0.985 (0.916–1.060)
Mother’s education level (year)	0.970 (0.900–1.045)	0.966 (0.897–1.039)
KIDMED score	0.878 (0.804–0.959) *	NI
Exercise (hours/week)	0.677 (0.557–0.823) **	NI
Clustering of unhealthy habits	NI	2.211 (1.536–3.182) **

NI not included. ** *p* < 0.001; * *p* = 0.004.

## Data Availability

The data presented in this study are available on request from the corresponding author. The data are not publicly available due to privacy reasons.
